# Enhancing Gene Delivery in NB-4 Cells: Overcoming Transduction and Selection Challenges

**DOI:** 10.3390/cells13221849

**Published:** 2024-11-08

**Authors:** Stefano Leto, Sonakshi Gehlot, Bhavwanti Sheth, Stefano Ratti, Lucia Manzoli, Nullin Divecha, Roberta Fiume

**Affiliations:** 1 Cellular Signalling Laboratory, Department of Biomedical Sciences, University of Bologna, 40126 Bologna, Italy; stefano.leto2@unibo.it (S.L.); stefano.ratti@unibo.it (S.R.); lucia.manzoli@unibo.it (L.M.); 2 Inositide Laboratory, School of Biological Sciences, Faculty of Environmental and Life Sciences, University of Southampton, Highfield, Southampton SO17 1BJ, UK; s.gehlot@soton.ac.uk (S.G.); b.sheth@soton.ac.uk (B.S.)

**Keywords:** gene transduction, NB-4 leukemia cells, viral vector engineering

## Abstract

Efficient gene transduction and cell viability are critical factors in genetic manipulation for research and therapeutic purposes. In this study, we explored the challenges associated with transducing the NB-4 cell line, a well-established model for acute promyelocytic leukemia (APL), using lentiviral vectors. While the initial transduction efficiency in NB-4 cells reached approximately 30%, we observed a significant decrease in cell viability, a phenomenon not observed in other acute leukemia cell lines such as THP-1 cells. We identified that this toxicity could be mitigated by purifying viral particles through ultracentrifugation or polyethylene glycol (PEG) precipitation, indicating that toxic substances, potentially secondary metabolites released by HEK293, could be responsible for the cell death. Nevertheless, cell selection by puromycin was still ineffective; crucially, we discovered that the human phosphoglycerate kinase (hPGK) promoter, commonly used in the PLKO1 vector, may become silenced in NB-4 cells, preventing effective selection with puromycin. By replacing the hPGK promoter with the elongation factor-1 alpha (EF1α) promoter, we successfully achieved high transduction efficiency and robust selection, demonstrating the potential for this modified vector system to facilitate genetic studies in APL models. These findings provide important insights into optimizing gene transduction protocols not only for NB-4 cells but also for other challenging cell lines, offering a refined approach for gene delivery and selection in cell models.

## 1. Introduction

Gene transduction, the process of introducing foreign genes into cells, is a fundamental technique in molecular biology with significant applications in both research and therapy. This method is essential for studying gene function and regulation and is crucial in creating genetically modified cell lines, which are pivotal for developing gene therapies. Beyond its research applications, gene transduction is instrumental in therapeutic strategies that require precise genetic manipulation in living organisms, highlighting its central role in advancing modern medicine [1,2].

However, the efficiency and effectiveness of gene transduction can vary significantly depending on the method used, the type of cells targeted, and whether viral or non-viral vectors are employed. For example, lentiviral vectors are highly effective for transducing both dividing and non-dividing cells, making them ideal for stem cell research. Adenoviral vectors, known for their high transduction efficiency, are commonly used in cancer gene therapy. However, their effectiveness can differ based on the cell type; for instance, some cells may resist transduction due to surface receptor variability. Non-viral methods, such as electroporation or liposome-mediated transfection, offer alternative approaches that can bypass issues like immune responses or cell-specific resistance, making them valuable for certain cell types, such as primary cells or neurons, which can be challenging to transduce using viral vectors [3,4,5]. Ex vivo transduction of cells is particularly important and useful in manipulating the cells of the immune system, such as hematopoietic stem cells, which can be easily collected, genetically modified, and then reintroduced into the patient. A prime example of this is the generation of CAR-T cells, where T cells are engineered ex vivo to express chimeric antigen receptors (CARs) that specifically target and destroy human tumors [6].

In leukemia research, the ability to transduce human or mouse cells with tools that manipulate gene expression is essential. This technique enables the reintroduction of genetically modified cells into mouse models, facilitating the exploration of disease development and the identification of critical molecular drivers. Such experiments are often key to uncovering novel therapeutic targets. For instance, by transducing cells to overexpress or silence specific genes, it becomes possible to observe the effects on leukemia progression, paving the way for the development of small-molecule inhibitors or CRISPR-based therapies [7,8].

The NB-4 cell line is a well-established model for promyelocytic leukemia research, known for harboring the characteristic t(15;17) translocation, which results in the PML-RARA fusion gene. This mutation is crucial in blocking the differentiation of promyelocytes, leading to the accumulation of immature cells that drive leukemia development. NB-4 cells have been instrumental in defining the first differentiation therapies for promyelocytic leukemia, specifically those based on all-trans retinoic acid (ATRA) and arsenic trioxide. These therapies exploit the ability of ATRA and arsenic acid to induce differentiation and apoptosis in promyelocytes, effectively transforming the management of the disease. As a result, NB-4 cells continue to be a valuable tool for studying the molecular mechanisms of promyelocytic leukemia and for developing and testing new therapeutic strategies [9,10].

While the differentiation strategy has indeed achieved a remarkable 95% cure rate for promyelocytic leukemia patients, a small subset of patients still experience relapse. This low percentage has unfortunately not been a strong enough motivator for the pharmaceutical industry to develop novel second-line therapies [11,12]. In our efforts to identify new targets for treating differentiation-resistant promyelocytic cells, we encountered significant obstacles in implementing a gene silencing approach using lentiviral vectors containing short hairpin RNA (shRNA) in NB-4 cells. Despite adhering to the established protocols, we consistently faced low transduction efficiency and poor cell survival post-transduction, which hindered effective gene knockdown [13].

To address these issues, we conducted a thorough evaluation of our viral transduction methods. We identified and resolved the underlying problems by employing viral precipitation techniques and utilizing a different promoter construct for improved selection. This new methodology for transduction not only enhances the effectiveness of experiments involving NB-4 cells but is also expected to benefit a wide range of cell lines used in gene transduction research.

## 2. Materials and Methods

### 2.1. Cell Lines

The THP-1 cell line was obtained from DMSZ (Braunschweig, Germany), while the NB-4 cell line was generously provided by Prof. Minucci, having been originally derived in the Pelicci laboratory. The HEK293FT cell line was purchased from Life Technologies, Carlsbad, CA, USA. All cell lines were cultured under standard conditions (5% CO_2_, 37 °C) in RPMI-1640 or DMEM (for HEK293FT) media, supplemented with 10% FBS, 10 U/mL penicillin, 10 µg/mL streptomycin, and 2 mM L-glutamine (Life Technologies). Cell line authentication was performed in-house using short tandem repeat (STR) analysis where reference patterns were available [14].

### 2.2. Lentivirus Production: Transduction and Spinoculation

**Figure 1:**
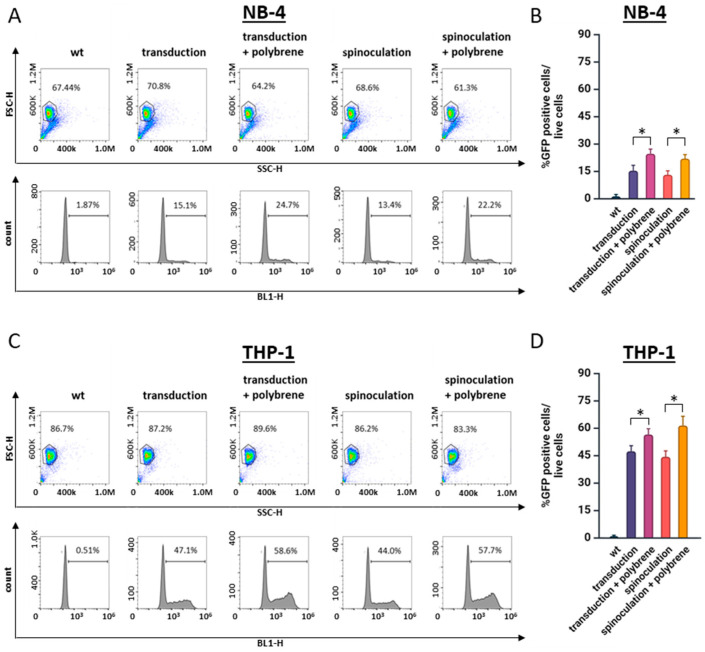
Comparison of the impact of spinoculation and treatment with polybrene on cell transduction efficiency. (**A**) NB-4 and (**C**) THP-1 cells were subject to either transduction or spinoculation, with or without the addition of polybrene. Results were evaluated 48 h post-transduction using flow cytometry. Cells within the gate in the dot plot represent the live cell population. The histograms display the percentages of GFP-positive cells, indicating successful transduction. Transduction efficiencies for NB-4 and THP-1 cells are shown in graphs (**B**) and (**D**), respectively. Statistical analysis was performed using one-way ANOVA, with * *p* < 0.05 considered significant when compared to the control.

**Figure 2:**
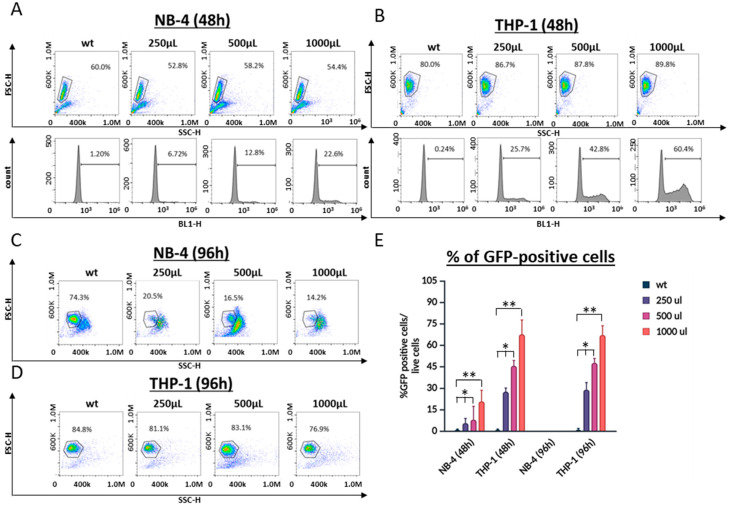
Comparison of the amount of virus on transduction efficiency. (**A**) NB-4 and (**B**) THP-1 cells transduced with increasing volumes of virus. Results were evaluated 48 h post-transduction using flow cytometry. Cells within the gate in the dot plot represent the live cell population. The histograms display the percentages of GFP-positive cells, indicating successful transduction. (**C**) NB-4 and (**D**) THP-1 cells were analyzed again 96 h post-transduction. Results demonstrate an increase in transduction efficiency with higher viral loads, with the efficiency remaining stable over time. Transduction efficiencies for NB-4 and THP-1 cells are shown in graph (**E**). Statistical analysis was performed using one-way ANOVA, with * *p* < 0.05 and ** *p* < 0.01 considered significant when compared to the control.

**Figure 3:**
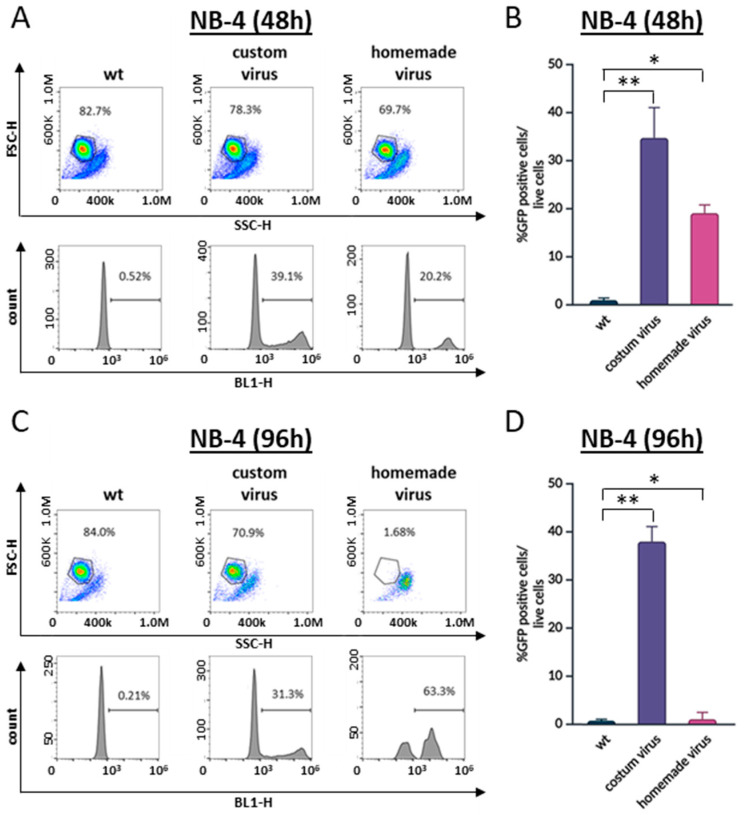
Comparison between in-house-produced and custom-made commercial virus preparations on cell transduction efficiency. NB-4 cells were transduced using custom-purified viral particles and laboratory-generated viral supernatants. Results were evaluated (**A**) 48 h and (**C**) 96 h post-transduction using flow cytometry. Cells within the gate in the dot plot represent the live cell population. The histograms display the percentage of GFP-positive cells, indicating successful transduction. (**A**) At 48 h post-transduction, only 20% of NB-4 cells were successfully transduced with the homemade virus, while 40% of the cells were transduced with the custom virus. Furthermore, (**C**) by 96 h post-transduction, NB-4 cells transduced with homemade virus had died, while cells transduced with custom virus remained viable. Transduction efficiencies at 48 h and 96 h are shown in graphs (**B**) and (**D**), respectively. Statistical analysis was performed using one-way ANOVA, with * *p* < 0.05 and ** *p* < 0.01 considered significant when compared to the control.

**Figure 4:**
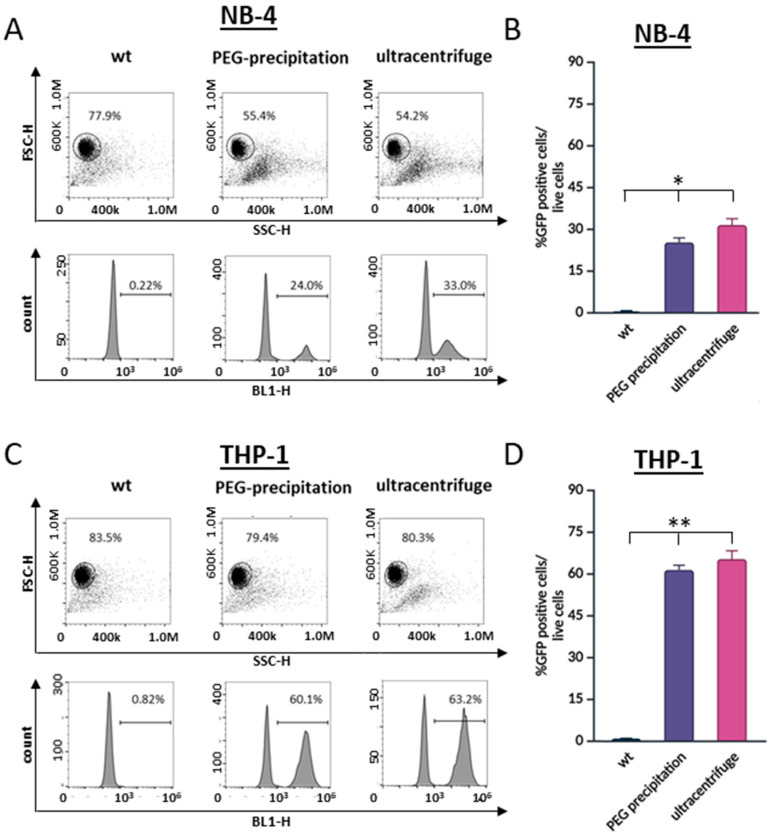
Comparison between PEG precipitation and ultracentrifugation for virus particle concentration on cell transduction efficiency. Viral particles were purified using either polyethylene glycol (PEG) precipitation or high-speed centrifugation to transduce (**A**) NB-4 and (**C**) THP-1 cells. Results were evaluated 48 h post-transduction using flow cytometry. Cells within the gate in the dot plot represent the live cell population. The histograms display the percentages of GFP-positive cells, indicating successful transduction. Transduction efficiencies for NB-4 and THP-1 cells are shown in graphs (**B**) and (**D**), respectively. Both methodologies demonstrated effectiveness in enhancing cell viability and increasing transduction percentages. Statistical analysis was performed using one-way ANOVA, with * *p*  <  0.05 and ** *p* < 0.01 considered significant when compared to the control.

**Figure 5:**
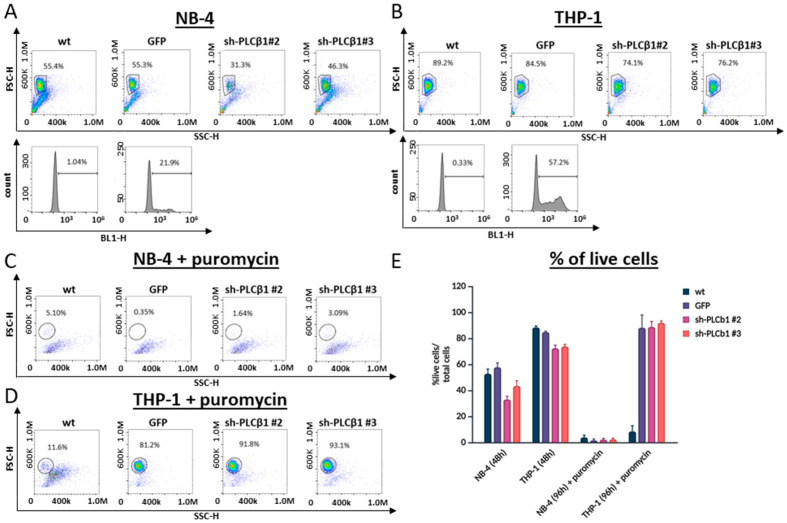
Evaluation of puromycin resistance of NB4 cells after viral transduction with purified concentrated virus. Cells were transduced using PEG-purified viruses encoding either GFP or 2 different shRNAs targeting PLCβ1 (but not containing GFP gene). 48 h post-transduction, (**A**) NB-4 and (**B**) THP-1 were analyzed by flow cytometry. Cells within the gate in the dot plot represent the live cell population. The histograms display the percentage of GFP-positive cells, indicating successful transduction. (**C**) NB-4 and (**D**) THP-1 cells were analyzed again 48 h after puromycin selection (96 h after transduction). The percentages of live cells are presented in graph (**E**). Transduced NB-4 cells did not survive puromycin selection, whilst THP-1 did.

**Figure 6:**
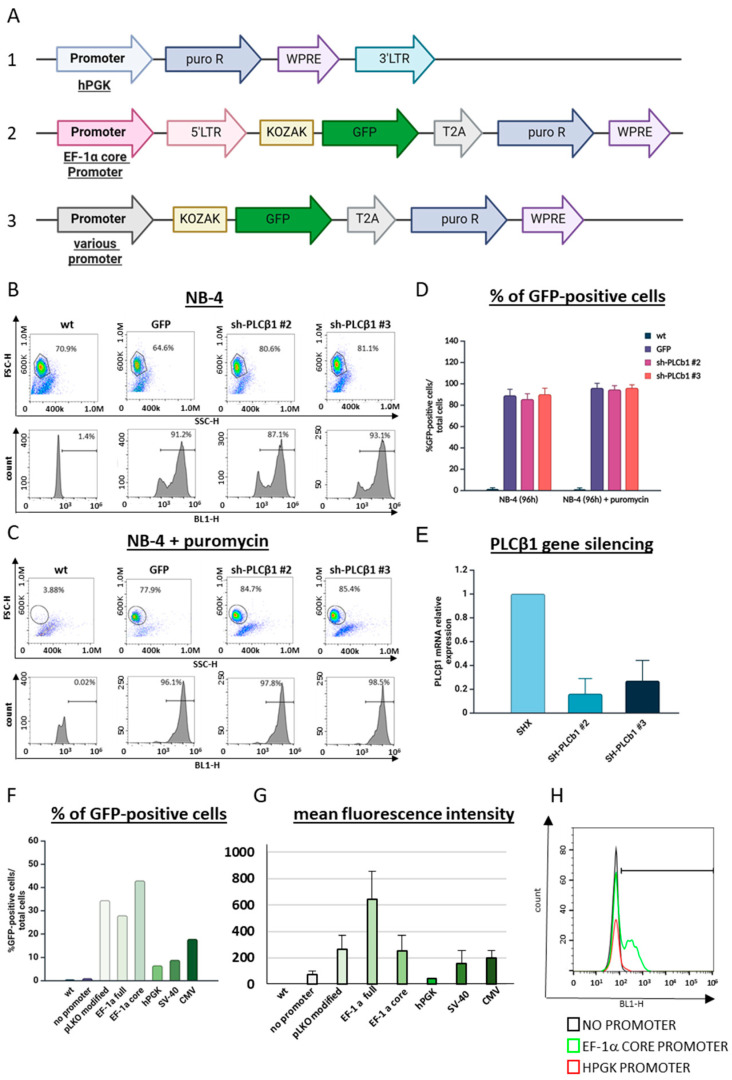
Evaluation of transduction efficiency in NB-4 cells using a newly engineered transduction vector. (**A**) 1. Puromycin selection cassette driven by the hPGK promoter in the PLKO vector was replaced with the GFP-P2A-puromycin cassette, driven by the EF1 promoter isolated from the pCDH vector to generate vector 2. 3 is a vector generated based on 2 to test different promoters for transgene expression in NB cells. Purified viral particles were generated using this new transfer vector with a non-targeting oligo (shx) and vectors encoding two shRNA oligos targeting PLCβ1; they were all used to transduce NB-4 cells. Results were analyzed by flow cytometry (**B**) 48 h after transduction and (**C**) 48 h after puromycin selection (96 h after transduction). Cells within the gate in the dot plot represent the live cell population. The histograms display the percentages of GFP-positive cells, indicating successful transduction. The corresponding transduction efficiency values are shown in graph (**D**). (**E**) RNA was isolated from the puromycin-selected cells, and qRT-PCR analysis of PLCβ1 expression was performed using actin as a housekeeping gene. Results show that with new vector NB-4 cells survived puromycin selection. (**F**) Different promoters (indicated in the graph) were cloned into vector 3 and used to generate purified virus, which was used to transduce NB4 cells. 48 h later, GFP expression was assessed using FAC analysis, and the % GFP-positive cells (**F**) and their mean florescence intensity (**G**) are plotted. A representative overlay of the empty promoter, the PGK promoter, and the EF1a promoter is shown (**H**) and demonstrates the very low average GFP expression from the PGK promoter compared to the EF1a promoter.

Lentiviral particles were produced by transfecting HEK293FT cells as outlined in Figure S1 and subsequently used to infect NB-4 and THP-1 cells. To determine the most efficient protocol for infecting hematopoietic cells with lentiviral particles, various conditions were tested, including different amounts of viral supernatant, virus particles after precipitation, the use of spinoculation, and the established use of polybrene. For all experiments, NB-4 and THP-1 cells were plated in six-well plates at a density of 0.5 × 10^6^ cells/mL in 2 mL of RPMI-1640 medium. Initially, 1 mL of virus-containing HEK293FT supernatant (as generated in Figure S1) was added to the cells in the absence or presence of polybrene (8 µg/mL). For transduction, virus was directly added to the cells, followed by overnight incubation at 37 °C. For spinoculation, cells were resuspended with viral supernatants and then centrifuged at low g (1100× *g*) for 30 min at room temperature. After centrifugation, cells were gently resuspended and 1 mL of fresh medium added, followed by incubation overnight at 37 °C. The following day, each well received an additional 2 mL of fresh RPMI-1640 medium (Figure 1). In subsequent experiments (Figure 2), different volumes of virus, prepared as described in Figure S1, were tested to optimize infection efficiency. For experiments shown in Figure 3, Figure 4, Figure 5 and Figure 6, 250 µL of concentrated virus was used, as described below. Where necessary, transduced cells were selected after an additional 24 h incubation by the addition of puromycin (1 µg/mL). For cells transduced with a Green Florescent Protein (GFP)-expressing transfer vector, transduction efficiency was assessed by flow cytometry (FACS). Data were analyzed by FlowJo software (FlowJo Inc. Version number 9).

### 2.3. NB-4 Cells Infection by Transduction or Spinoculation

To determine the most efficient protocol for infecting hematopoietic cells with lentiviral particles, various conditions were tested, including different amounts of viral supernatant, virus particles after precipitation, the use of spinoculation, and the established use of polybrene. Cells were plated in six-well plates at a density of 0.5 × 10^6^ cells/mL in 2 mL of RPMI-1640 medium. A varying amount of filtered or precipitated virus was added to the cells in the absence or presence of polybrene (8 µg/mL). Cells were either incubated overnight at 37 °C or subjected to spinoculation by centrifugation at 2200 rpm (g value to be determined) for 30 min at room temperature. After centrifugation, cells were gently resuspended by pipetting, and 1 mL of fresh medium was added. Cells were then incubated overnight at 37 °C, followed by the addition of 2 mL of fresh RPMI-1640 to each well the next day. Where necessary, transduced cells were selected after an additional 24 h incubation by the addition of puromycin (1 µg/mL). For cells transduced with a GFP-expressing transfer vector, transduction efficiency was assessed by flow cytometry (FACS).

### 2.4. Virus Concentration by Ultracentrifugation or Polyethylene Glycol (PEG) Precipitation

To concentrate and purify the virus, two methods were employed. For ultracentrifugation, the filtered viral supernatant was centrifuged at 100,000× *g* for 60 min. The supernatant was then carefully removed, and the viral pellet was resuspended in RPMI-1640 medium over a period of 4 h. Further, 6 mL of virus-containing HEK293FT supernatant was resuspended after ultracentrifuging in 1 mL of fresh RPMI. Resuspended virus was either used immediately or aliquoted into 250 µL portions and stored at –80 °C.

For PEG precipitation, a 4X PEG-8000 solution was prepared by dissolving 80 g PEG-8000 and 14.0 g NaCl in 80 mL Milli-Q water, followed by the addition of 20 mL 10X PBS (pH 7.4). The pH was adjusted to 7.0–7.2, and the final volume was brought to 200 mL before sterile filtration through a 0.2 µm filter. This PEG solution was added to the viral supernatant at a volume of 1:3 (PEG solution: virus), vortexed briefly, and incubated overnight at 4 °C with gentle agitation. The virus was then pelleted by centrifugation at 1600× *g* for 60 min at 4 °C. The supernatant was discarded, and the viral pellet was resuspended in RPMI-1640 at a volume equal to one-sixth of the original viral supernatant.

### 2.5. Cloning

Initially, we used a TRC2 pLKO shRNA vector (from Sigma-Aldrich, St. Louis, MO, USA) negative control (wt) containing GFP gene or two different oligo to silence phospholipase C beta 1 (PLCb1). To construct the vector sh-EF1-COP-T2A-Puro, a PCR fragment was amplified from the pCDH_EF1_COP_T2A_puro plasmid using the primers EF1A_GFP_PURO_slice_F2 and rev_cop_puro_slice (Supplementary Table S1). This fragment contained the EF1α core promoter driving the expression of COP-GFP fused to the puromycin resistance gene via a T2A viral cleavage sequence. 
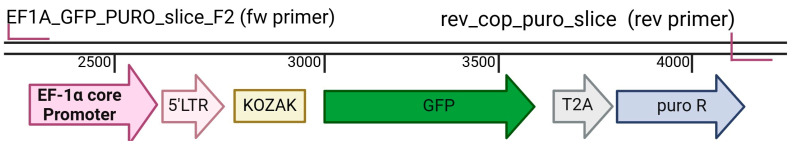


The PCR product was then inserted into the TRC-2 shRNA vector, replacing the HPGK promoter that originally drove the puromycin resistance gene. For this replacement, the vector was digested with XhoI and MluI, and the PCR fragment was inserted using SLiCE-mediated homologous recombination. The resulting construct, sh-EF1-COP-T2A-Puro, enables the expression of shRNA along with COP-GFP and puromycin resistance under the EF1α promoter. For subsequent experiments, shRNA oligos previously cloned into the TRC-2 vector were transferred into this new construct using the NotI and EcoRI sites. Additionally, shRNA oligos originally cloned into the TRC1 vector can be transferred into sh-EF1-COP-T2A-Puro using the NdeI and EcoRI restriction sites.

To generate a vector suitable for testing promoter efficiency, we modified the sh-EF1-COP-T2A-Puro vector by removing the EF1α core promoter. Additionally, we removed the truncated 5′ LTR sequence from the pCDH vector, which had been incorporated into sh-EF1-COP-T2A-Puro, as we hypothesized that this element could influence transcriptional activity. These modifications were performed using reverse PCR with primers designed to introduce SpeI and NheI sites on the forward strand using the primer F_promoter_test_spe1_F and SpeI and BamHI sites on the reverse strand using the primer F_promoter_test_spe1_R (see Supplementary Table S1). After PCR amplification, we digested the ends with SpeI and ligated them to create the modified pProm_test plasmid, enabling directional cloning of promoters into the BamHI-NheI sites. We then generated PCR fragments encoding the EF1α core promoter (matching the promoter in sh-EF1-COP-T2A-Puro), the full EF1α promoter, the SV40 promoter, the CMV+enhancer promoter, and the HPGK1 promoter (as in the shPLKO plasmid) using the listed primers (Supplementary Table S1 to generate the sequences listed in supplentary_fasta file_promoter_sequences). Each promoter was digested with BamHI and NheI, then cloned into the digested and dephosphorylated pProm_test plasmid. Following sequence verification, promoter plasmids were purified for viral production. To produce virus, we transfected HEK293 cells in six-well plates and collected 2 mL of viral supernatant on days 1, 2, and 3. The 6 mL of viral supernatant was concentrated to 200 µL by centrifugation at 100,000× *g* for 1 h. A total of 75 µL of concentrated virus was used to transduce NB4 cells.

## 3. Results

### 3.1. Transduction and Spinoculation

To identify novel regulators of promyelocytic cell growth, we aimed to employ a knockout approach using a shRNA library targeting the signal transduction modulators in the NB-4 promyelocytic cell line. NB-4 cells are highly responsive to ATRA-induced differentiation; however, resistant cell lines can be generated through prolonged exposure to ATRA, providing a valuable model for exploring potential second-line therapies for relapsed promyelocytic leukemia patients. To facilitate this, we first assessed the transduction efficiency of NB-4 cells using viral transduction protocols previously established for other cell lines. As a control, we utilized THP-1 cells, which are a well-characterized monocytic cell line known for their robust transduction efficiency with lentiviral vectors [15]. Spinoculation, or centrifugal enhancement, employs low centrifugal force (~1000× *g*) to improve viral uptake and has been widely used to enhance the infection efficiency for various viruses, including HIV-1, on which the lentiviral system in this study is based [16,17,18,19]. While the precise mechanism remains unclear, spinoculation appears to increase the binding of viral particles to cells [18], although the applied force is typically thought to be insufficient to directly sediment the viral particles. Recent findings suggest that spinoculation also activates cofilin in the host cell, which triggers actin dynamics favorable for viral entry, particularly in HIV-1 infections of T cells [20]. Interestingly, studies show that spinoculating the cells before the viral addition does not enhance the subsequent infection, implying that any increase in actin dynamics needs to occur in the immediate presence of viral particles. Spinoculation has proven to be especially effective in transducing hematopoietic cells and other cells maintained in suspensions, which are generally more resistant to infection.

The transfection efficiency in the HEK293 cells consistently ranged from 50% to 80% across all the experiments, indicating robust viral production and high titers. To optimize the transduction in the NB-4 cells, we compared two techniques, standard transduction and spinoculation, and evaluated the effect of polybrene, a cationic polymer that enhances viral infection by neutralizing the charge repulsion between the virions and sialic acid on the cell surface [21,22]. Following transduction, the cells were analyzed by flow cytometry (FACS) at 48 and 72 h post-infection. The live cell population was gated based on forward and side scatter, with the transduction efficiency assessed within this R1-gated population. In the NB-4 cells, the transduction efficiency without polybrene was approximately 15%, which increased to 24% with the polybrene treatment (Figure 1A). However, this was significantly lower compared to the THP-1 cells, where the transduction efficiency was 47% without polybrene and 60% with polybrene (Figure 1B). Interestingly, spinoculation, a technique commonly used to enhance viral transduction in hematopoietic cells such as THP-1, did not improve the transduction efficiency in either the NB-4 or THP-1 cells regardless of the presence or absence of polybrene (Figure 1A,B).

Given the low transduction efficiency observed in the NB-4 cells, we investigated whether increasing the viral load could enhance the transduction efficiency. We varied the viral load by adjusting the amount of viral supernatant added, conducting these experiments in the presence of polybrene but without spinoculation. Both the NB-4 and THP-1 cells showed dose-dependent increases in transduction efficiency; however, in the NB-4 cells, this increase only reached 22% with 1 mL of viral supernatant (Figure 2A,B).

While further increasing the viral load was considered, we observed a dramatic decline in cell viability to 14% at 96 h post-transduction when 1 mL of viral supernatant was used. Interestingly, this decrease in viability was not observed in the THP-1 cells, suggesting that NB-4 cells may be particularly sensitive to components within the viral supernatant (Figure 2C,D).

### 3.2. Purification of Viral Particles by Ultracentrifugation and PEG Precipitation

To assess whether the viral supernatant might be toxic to NB-4 cells, we compared the transduction efficiency using custom-purified viral particles versus a laboratory-generated viral supernatant. Transduction with the purified viral particles resulted in approximately 70% efficiency, whereas the viral supernatant yielded only 22%, consistent with the previous observations (Figure 3A). Notably, the transduction with the viral supernatant led to a significant decrease in cell viability at 96 h, while the transduction with the purified viral particles did not result in any cell death (Figure 3B). This suggests that components present in the viral supernatant, which can be removed by the purification of the viral particles, may be responsible for the observed toxicity in NB-4 cells.

We next evaluated two established methods for purifying viral particles: high-speed centrifugation and polyethylene glycol (PEG) precipitation [23]. For transduction, we used equivalent amounts of purified virus corresponding to 1 mL of the viral supernatant. Both the PEG-precipitated and centrifuged virus achieved approximately 30% transduction efficiency in the NB-4 cells and about 60% in the THP-1 cells (Figure 4A,B). Crucially, at 96 h post-transduction, there was only a slight decrease in NB-4 cell viability. Given that both PEG precipitation and ultracentrifugation produced efficient virus preparations that did not induce cell death, we opted to proceed with PEG precipitation due to its simplicity and the lack of specialized equipment required.

### 3.3. Target Gene Silencing by Transduction with Purified Virus Generated from a Novel Transfer Plasmid

Given that most shRNA or sgRNA libraries can be selected using puromycin, we considered a 30% transduction efficiency sufficient for further selection to achieve 100% transduced cells. To test this, we generated PEG-purified virus from a GFP transfer plasmid encoding puromycin resistance, as well as two additional transfer plasmids encoding shRNA targeting phospholipase Cβ1, a key enzyme involved in GPCR and nuclear polyphosphoinositide (PPIn) signaling, both of which also conferred puromycin resistance (but did not have GFP genes).

As previously demonstrated, the transduction efficiency with the GFP virus reached approximately 22% in the NB-4 cells and 58% in the THP-1 cells (Figure 5A,B). After 48 h, the cells were treated with puromycin (1 µg/mL) and incubated for an additional 48 h. As expected, the percentage of GFP-positive THP-1 cells increased to nearly 100%, and those cells transduced with the shRNA vectors exhibited over 90% viability, indicating successful transduction and selection (Figure 5D). In stark contrast, the selection of transduced NB-4 cells with puromycin resulted in 99% cell death despite 22% of the NB-4 cells being GFP-positive prior to selection (Figure 5C). These results suggest that the HPGK promoter driving puromycin resistance may be silenced in NB-4 cells but remains active in THP-1 cells. To test this hypothesis, we generated purified viral particles using a pCDH1 transfer vector, in which GFP expression is driven by an EF1 promoter and puromycin resistance is linked via a T2A cleavage site fused to a GFP. Remarkably, transduction with these purified viral particles resulted in approximately 30% GFP-positive NB-4 cells, which could be efficiently selected to nearly 100% using puromycin. These findings strongly support the hypothesis that the human PGK promoter may become silenced in NB-4 cells following transduction, but not in THP-1 cells.

To confirm this and create a more reliable vector for shRNA or sgRNA transduction in NB-4 cells, we replaced the puromycin selection cassette in the PLKO vector with the GFP-P2A-puromycin cassette from pCDH1, driven by the EF1 promoter (sh-EF1-COP-T2A-Puro) (Figure 6A, vector 2). Purified viral particles were generated using this new transfer vector with a non-targeting oligo, as well as vectors encoding two shRNA oligos targeting PLCβ1. The resulting viral particles showed high efficiency in transducing NB-4 cells, with approximately 70–80% of the cells becoming GFP-positive (Figure 6B). Importantly, puromycin selection led to nearly 100% of the cells expressing GFP across all three transfer vectors, while the non-transduced cells were completely eliminated by puromycin (Figure 6C). To further validate the effectiveness of this new vector in gene knockdown, RNA was isolated from the puromycin-selected cells and qRT-PCR analysis of the PLCβ1 expression was performed using actin as a housekeeping gene. Both shRNAs targeting PLCβ1 reduced its expression to less than 20% compared to the non-targeting shRNA construct, demonstrating the vector’s efficiency in gene silencing in NB-4 cells (Figure 6D).

Our data suggest that the puromycin resistance cassette driven by the PGK promoter shows low or silenced expression compared to the EF1α core promoter. However, these promoters were not tested under identical conditions, making direct comparisons challenging. To address this, we re-cloned various promoters commonly used for mammalian expression into the same vector, replacing the EF1α core promoter and removing the 5′ LTR (Figure 6A, vector 3). This step eliminated the LTR’s influence on the transcriptional output as LTR elements are known to modulate transcription. For consistency, the EF1α core promoter was reinserted into the modified vector as a control. Additionally, we cloned the EF1 full promoter, SV40, CMV-enhancer, and human PGK (the same as the one utilized in the PLKO-1 vector) promoters into this vector and used them to test the level of GFP expression in the NB4 cells. The promoter insertions were confirmed by sequencing, and purified virus was produced after transfection into HEK293. The percentages of HEK cells transfected were similar in all the cases. The NB4 cells were then infected without polybrene or spinoculation, and the GFP expression was analyzed by FACS two days post-transduction (Figure 6F). As expected, those cells transduced with a vector lacking any promoter showed no GFP expression, while those with the EF1α core promoter and LTR achieved ~30% transduction (sh-EF1-COP-T2A-Puro). Notably, removing the LTR and retaining only the EF1α core promoter yielded similar GFP expression, suggesting that the LTR does not significantly impact transcription in this context.

Further analysis revealed that the full EF1α promoter enhanced the mean fluorescence intensity without altering the proportion of GFP-positive cells. In contrast, the vectors with the CMV-enhancer or SV40 promoters showed substantially lower GFP expression, indicating that the viral promoters may be silenced in NB4 cells. Consistent with our observations, the PGK promoter yielded few GFP-positive cells with a low mean fluorescence intensity (Figure 6F–H). These results support our hypothesis that the PGK promoter is silenced in NB4 cells and highlight the EF1α promoter as a favorable option for the transgene expression in this line.

## 4. Discussion

The primary challenges encountered in this study were the low transduction efficiency and reduced cell survival following the viral infection of NB-4 cells. Consistent with previous reports, the inclusion of polybrene during the transduction process enhanced the efficiency. However, surprisingly, spinoculation, commonly employed to boost the transduction efficiency in hematopoietic cells, did not yield any significant improvement in either THP1 or NB-4 cells. The mechanism by which low-speed centrifugation (spinoculation) enhances viral infection remains incompletely understood, although it is hypothesized that the process may create a concentration gradient or stimulate the endocytic pathways, thereby facilitating viral entry into the cells. Despite achieving a transduction efficiency of approximately 30% in NB-4 cells, we observed a significant decrease in cell viability following transduction with viral supernatants. This reduction in viability was specific to NB-4 cells as it did not occur in THP1 cells and could be mitigated in NB-4 cells by purifying the viral particles through ultracentrifugation or PEG precipitation. This finding suggests that a toxic substance, potentially released by HEK293 cells during viral production, is affecting NB-4 cells. While endotoxins from the purified DNA used for transfection or PEI used in the transfection process are plausible candidates, we believe these are unlikely culprits since the cell supernatant containing most of these substances is replaced with fresh medium after transfection. A more probable explanation is the release of secondary metabolites from HEK293 cells when grown at high densities to generate high viral titers, which may be particularly toxic to NB-4 cells. This issue appears to be specific to NB-4 cells as we have successfully transduced various human cell lines and primary cells, including human T cells, using non-purified viral supernatants without observing similar toxicity. It is noteworthy that studies involving the transduction of NB-4 cells typically employ purified virus, often to concentrate the viral particles, although our findings suggest that purification also serves to mitigate cell toxicity.

A 30% transduction efficiency would be adequate for shRNA targeting when combined with puromycin selection for transduced cells. However, further selection with puromycin after transduction with the PLKO1 vector proved to be unsuccessful, highlighting a potential critical issue in the transduction of NB-4 promyelocytic leukemia cells using lentiviral vectors, particularly when relying on the HPGK promoter for puromycin selection. The finding that those NB4 cells transduced with PLKO1—a lentiviral vector using the HPGK promoter to drive puromycin resistance—failed to become puromycin-resistant while the cells transduced with the pCDH vector, which uses the EF1α core promoter, showed strong resistance indicates that the HPGK-driven expression is either low or silenced in NB4 cells. Transgene silencing is a frequent occurrence in viral transduction and poses a significant limitation in recombinant protein production for therapeutic applications, which rely on stable transgene expression [24]. Silencing is also a challenge in cell reprogramming. Host cells employ several mechanisms to silence transgenes, including DNA methylation [25], chromatin modifications [26,27,28], and the recruitment of sequence-specific restriction factors for viral sequences [29,30]. Silencing can also occur if viral integration happens within heterochromatin regions, near telomeres, or in areas subject to transcriptional repression. One approach to counteract silencing involves targeting transgenes to open chromatin regions, such as “safe harbor” sites, or using insulators to block the spread of heterochromatin. However, even in these cases, silencing can still occur [24]. Using endogenous promoters instead of viral promoters like SV40 or CMV can also reduce silencing. For instance, previous research has shown that certain promoters, including CMV and SV40, are prone to silencing in stem cells and primary cells, leading to reduced transgene expression over time [31]. Similarly, studies in other leukemia cell lines have demonstrated that the activity of viral promoters can be significantly diminished, necessitating the use of alternative promoters like EF1 or CAG, which are less susceptible to silencing and maintain more consistent expression. In keeping with the data presented here, previous studies using commonly used promoters to drive GFP expression showed that, in many cell lines, the PGK promoter results in lower expression than EF1α or CMV promoters [32]. The mechanism of silencing of the PGK promoter observed in NB4 cells remains unclear and warrants further investigation to characterize the epigenetic landscape surrounding the promoter. Identifying the specific epigenetic factors at play could provide insight into why the HPGK promoter is particularly susceptible to silencing in this cell line. The finding that the transgene expression driven by the EF1α promoter is robust enough to confer puromycin resistance and drive GFP expression is valuable for other researchers and may have significant implications for transgene therapy in AML. Our observations are consistent with other studies that have reported promoter silencing as a barrier in various cell types, particularly in hematopoietic and cancer cells, where the chromatin structure and epigenetic modifications can influence the promoter activity [33,34].

## 5. Conclusions

Our study contributes to this body of knowledge by providing a practical solution for overcoming promoter silencing in NB-4 cells, a model system widely used for studying acute promyelocytic leukemia (APL). The newly developed vector, sh-EF1-COP-T2A-Puro, not only enhanced the transduction efficiency but also enabled robust gene knockdown, as demonstrated by the significant reduction in PLCβ1 expression. This vector could be of considerable value in future studies aimed at exploring the gene functions, drug resistance mechanisms, and therapeutic targets in NB-4 cells and other difficult-to-transduce cell lines and underscores the importance of optimizing the viral vector design for successful gene transduction strategies of specific cell types.

## Data Availability

Data are contained within the article and Supplementary Materials.

## Supplementary Materials

The following supporting information can be downloaded at: https://www.mdpi.com/article/10.3390/cells13221849/s1, Figure S1: Protocol for Lentivirus Generation Using HEK293 Cells; Supplementary fasta file promoter sequences. Table S1: primers used for cloning.
