# Supplementary material for: Enhancing Gene Delivery in NB-4 Cells: Overcoming Transduction and Selection Challenges
Source: Cells. 2024 Nov 8;13(22):1849. doi: 10.3390/cells13221849 (PMC11593156; doi:10.3390/cells13221849)
Supplement: Supplementary file 1 [file cells-13-01849-s001.zip › cells-3221884-supplementary.pdf]

Supplementary files (3 files):

1: Figure S1

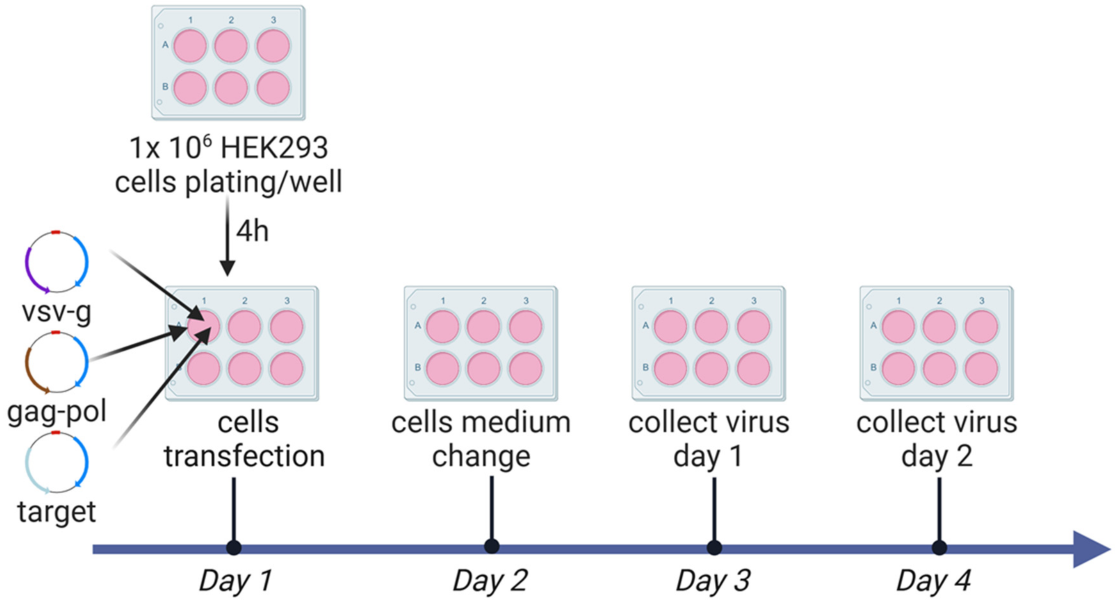

Figure S1: Protocol for Lentivirus Generation Using HEK293 Cells. HEK293 cells are seeded in a 6-well plate at a density of 1 x 10^6 cells per well in 2 mL of DMEM. Approximately 3-4 hours post-seeding, cells are transfected with a vector mix containing three plasmids required for lentivirus production: VSV-G, Gag-Pol, and the target expression vector (Day 1). On Day 2, the medium is replaced with 2 mL of fresh DMEM. Viral supernatants are collected on Days 3 and 4 for downstream applications.

2: Supplementary List: Supplementary fasta file promoter sequences

>Ef1a_core
GGGCAGAGCGCACATCGCCCACAGTCCCCGAGAAGTTGGGGGGAGGGGTCGGCAATTGAA
CGGGTGCCTAGAGAAGGTGGCGCGGGGTAAACTGGGAAAGTGATGTCGTGTACTGGCTCCG
CCTTTTTCCCGAGGGTGGGGGAGAACCGTATATAAGTGCAGTAGTCGCCGTGAACGTTCTTT
TTCGCAACGGGTTTGCCGCCAGAACACAG
>CMV_enhancer
TTCCGCGTTACATAACTTACGGTAAATGGCCCGCCTGGCTGACCGCCCAACGACCCCCGCCC
ATTGACGTCAATAATGACGTATGTTCCCATAGTAACGCCAATAGGGACTTTCCATTGACGTCA
ATGGGTGGAGTATTTACGGTAAACTGCCCACTTGGCAGTACATCAAGTGTATCATATGCCAA
GTACGCCCCCTATTGACGTCAATGACGGTAAATGGCCCGCCTGGCATTATGCCCAGTACATG
ACCTTATGGGACTTTCCTACTTGGCAGTACATCTACGTATTAGTCATCGCTATTACCATGGTGA
TGCGGTTTTGGCAGTACATCAATGGGCGTGGATAGCGGTTTGACTCACGGGGATTTCCAAGT
CTCCACCCCATTGACGTCAATGGGAGTTTGTTTTGGCACCAAAATCAACGGGACTTTCCAAA
ATGTCGTAACAACTCCGCCCCATTGACGCAAATGGGCGGTAGGCGTGTACGGTGGGAGGTC
TATATAAGCAGAGCTGGTTTAG
>SV40_promoter
GGTGTGGAAAGTCCCCAGGCTCCCCAGCAGGCAGAAGTATGCAAAGCATGCATCTCAATTA
GTCAGCAACCAGGTGTGGAAAGTCCCCAGGCTCCCCAGCAGGCAGAAGTATGCAAAGCAT
GCATCTCAATTAGTCAGCAACCATAGTCCCGCCCCTAACTCCGCCCATCCCGCCCCTAACTC
CGCCCAGTTCCGCCCATTCTCCGCCCCATGGCTGACTAATTTTTTTTATTTATGCAGAGGCCG
AGGCCGCCTCGGCCTCTGAGCTA
>EF1a _Full

GTGCCCGTCAGTGGGCAGAGCGCACATCGCCCACAGTCCCCGAGAAGTTGGGGGGAGGGG
TCGGCAATTGAACCGGTGCCTAGAGAAGGTGGCGCGGGGTAAACTGGAAAGTGATGTCGT
GTACTGGCTCCGCCTTTTTCCCGAGGGTGGGGGAGAACCGTATATAAGTGCAGTAGTCGCCG
TGAACGTTCTTTTTCGCAACGGGTTTGCCGCCAGAACACAGGTAAGTGCCGTGTGTGGTTCC
CGCGGGCCTGGCCTCTTTACGGGTTATGGCCCTTGCGTGCCTTGAATTACTTCCACCTGGCTG
CAGTACGTGATTCTTGATCCCGAGCTTCGGGTTGGAAGTGGGTGGGAGAGTTCGAGGCCTTG
CGCTTAAGGAGCCCCTTCGCCTCGTGCTTGAGTTGAGGCCTGGCCTGGGCGCTGGGGCCGC
CGCGTGCGAATCTGGTGGCACCTTCGCGCCTGTCTCGCTGCTTTCGATAAGTCTCTAGCCATT
TAAAATTTTTGATGACCTGCTGCGACGCTTTTTTTCTGGCAAGATAGTCTTGTAAATGCGGGC
CAAGATCTGCACACTGGTATTTCGGTTTTTGGGGCCGCGGGCGGCGACGGGGCCCGTGCGTC
CCAGCGCACATGTTCGGCGAGGCGGGGCCTGCGAGCGCGGCCACCGAGAATCGGACGGGG
GTAGTCTCAAGCTGGCCGGCCTGCTCTGGTGCCTGGCCTCGCGCCGCCGTGTATCGCCCCGC
CCTGGGCGGCAAGGCTGGCCCGGTCGGCACCAGTTGCGTGAGCGGAAAGATGGCCGCTTC
CCGGCCCTGCTGCAGGGAGCTCAAAATGGAGGACGCGGCGCTCGGGAGAGCGGGCGGGTG
AGTCACCCACACAAAGGAAAAGGGCCTTTCCGTCCTCAGCCGTCGCTTCATGTGACTCCAC
GGAGTACCGGGCGCCGTCCAGgcacctcgattagttcttgagcttttggagtacgtCGTCTTTAGGTTGGGGGGA
GGGGTTTTATGCGATGGAGTTTCCCCACACTGAGTGGGTGGAGACTGAAGTTAGGCCAGCTT
GGCACTTGATGTAATTCTCCTTGGAATTTGCCCTTTTTGAGTTTGGATCTTGGTTCATTCTCAA
GCCTCAGACAGTGGTTCAAAGTTTTTTTCTTCCATTTCAGGTGTCGTGA
>HPGK_promoter
TTGCGCCTTTTCCAAGGCAGCCCTGGGTTTGCGCAGGGACGCGGCTGCTCTGGGCGTGGTTC
CGGGAAACGCAGCGGCGCCGACCCTGGGTCTCGCACATTCTTCACGTCCGTTCGCAGCGTC
ACCCGGATCTTCGCCGCTACCCTTGTGGGCCCCCCGGCGACGCTTCCTGCTCCGCCCCTAAG
TCGGGAAGGTTCCTTGCGGTTCGCGGCGTGCCGGACGTGACAAACGGAAGCCGCACGTCTC
ACTAGTACCCTCGCAGACGGACAGCGCCAGGGAGCAATGGCAGCGCGCCGACCGCGATGG
GCTGTGGCCAATAGCGGCTGCTCAGCAGGGCGCGCCGAGAGCAGCGGCCGGGAAGGGGCG
GTGCGGGAGGCGGGGTGTGGGGCGGTAGTGTGGGCCCTGTTCCTGCCCGCGCGGTGTTCCG
CATTCTGCAAGCCTCCGGAGCGCACGTCGGCAGTCGGCTCCCTCGTTGACCGAATCACCGA
CCTCTCTCCCCAG

3: Table S1: primers used for cloning:

primers for cloning sh-EF1-COP-T2A-Puro
EF1A_GFP_PURO_slice_F2 ,CAGCAGAGATCCACTTTGGCGCCGGTGGGCAGAGCGCACATCG
CCCAC,,
GTTGATTGTCGACTTAACGCGTtcaggcaccgggcttgcgggtca

primers to generate pPROM_Test
F_promoter_test_spe1_F,gcttacctACTAGTGCTAGCCAAGCTGTGACCGGCGCCTACGC,,
rev_promoter_test_spe1_R,gattcccaACTAGTactggtcgagGGATCCCGCCAAAGTGGATCTCTGCT,,

primers for cloning various promoters
Forward,Reverse,mwt
SV40,GATCCTCGGATCCtgtgtgtcagttagggtgtgg,CAGCTTGGCTAGCcttctggaatagctcagaggcc,269
CMV_Enhancer,GATCCTCGGATCCGACCGCCATGTTGACATTGATTATTGACTAG,CAGCTTGG
CTAGCCGAGCTCTGCTTATATAGACCTCCC,598
HPGK,GATCCTCGGATCCTTGCGCCTTTTCCAAGGCAGC,CAGCTTGGCTAGCCCCTGGGGAG
AGAGGTCGGTG,511
EF1a_FULL,GATCCTCGGATCCGTGCCCGTCAGTGGGCAGAGC,CAGCTTGGCTAGCTCACGA
CACCTGAAATGGAAG,1172
EF1a_core,GATCCTCGGATCCGGTGGGCAGAGCGCACATCGC,CAGCTTGGCTAGCGCTGTGTT
CTGGCGGCAAAC,212
